# Oral Health Access Among Ethnic Minority Older Adults in the United States

**DOI:** 10.1093/geroni/igaf122.3631

**Published:** 2025-12-31

**Authors:** Xiaochuan Wang, Jasleen Sodhi

**Affiliations:** University of Central Florida, Orlando, Florida, United States; University of Central Florida, Orlando, Florida, United States

## Abstract

Oral healthcare is a critical yet often overlooked component of overall health among U.S. adults. Ethnic minority older adults, in particular, are disproportionately affected by poor oral health and related health conditions (e.g., cardiovascular disease and diabetes). These populations also experience significant disparities in access to dental care and preventive services, which can exacerbate existing health inequities. The present study aimed to systematically examine and synthesize existing literature to assess the extent to which oral health disparities exist among ethnic minority older adults in the U.S. and explore the roles of socioeconomic factors on these disparities. Guided by the Preferred Reporting Items for Systematic Reviews and Meta-Analyses (PRISMA) framework, we conducted a systematic review of peer-reviewed articles published in English between 2015 and 2025. Searches were conducted in electronic databases including PubMed, ProQuest, and EBSCOhost. The initial search yielded 464 unduplicated articles. After excluding articles based on title and abstract screening, 75 articles were reviewed in full text. Of these, a total of 59 articles met the inclusion criteria and were included in the qualitative synthesis. Across these studies, three primary barriers to oral healthcare among ethnic minority older adults were identified, including: systemic inequities in oral healthcare, cultural and linguistic barriers between providers and patients, and limited education and access specific to this population. These results underscore the need to improve access to care, implement culturally responsive provider training programs, and develop targeted educational resources to address oral health disparities among this underserved population.

